# VSNL1 Co-Expression Networks in Aging Include Calcium Signaling, Synaptic Plasticity, and Alzheimer’s Disease Pathways

**DOI:** 10.3389/fpsyt.2015.00030

**Published:** 2015-03-09

**Authors:** Chien-Wei Lin, Lun-Ching Chang, George C. Tseng, Caitlin M. Kirkwood, Etienne L. Sibille, Robert A. Sweet

**Affiliations:** ^1^Department of Biostatistics, University of Pittsburgh, Pittsburgh, PA, USA; ^2^Department of Psychiatry, University of Pittsburgh, Pittsburgh, PA, USA; ^3^Campbell Family Mental Health Research Institute, Centre for Addiction and Mental Health (CAMH), Departments of Psychiatry and Pharmacology & Toxicology, University of Toronto, Toronto, ON, Canada; ^4^Department of Neurology, University of Pittsburgh, Pittsburgh, PA, USA; ^5^VISN 4 Mental Illness Research, Education and Clinical Center (MIRECC), VA Pittsburgh Healthcare System, Pittsburgh, PA, USA

**Keywords:** visinin-like 1, visinin-like protein 1, Alzheimer disease, co-expression networks, calcium signaling, synaptic plasticity

## Abstract

The visinin-like 1 (VSNL1) gene encodes visinin-like protein 1, a peripheral biomarker for Alzheimer disease (AD). Little is known, however, about normal VSNL1 expression in brain and the biologic networks in which it participates. Frontal cortex gray matter obtained from 209 subjects without neurodegenerative or psychiatric illness, ranging in age from 16 to 91, was processed on Affymetrix GeneChip 1.1 ST and Human SNP Array 6.0. VSNL1 expression was unaffected by age and sex, and not significantly associated with SNPs in cis or trans. VSNL1 was significantly co-expressed with genes in pathways for calcium signaling, AD, long-term potentiation, long-term depression, and trafficking of AMPA receptors. The association with AD was driven, in part, by correlation with amyloid precursor protein (APP) expression. These findings provide an unbiased link between VSNL1 and molecular mechanisms of AD, including pathways implicated in synaptic pathology in AD. Whether APP may drive increased VSNL1 expression, VSNL1 drives increased APP expression, or both are downstream of common pathogenic regulators will need to be evaluated in model systems.

## Introduction

Alzheimer disease (AD) is the most prevalent form of dementia in the United States. It is characterized clinically by declining memory, progressive loss of cognitive ability, and behavioral changes. The incidence of AD increases rapidly with increasing age (1). This suggests a role of brain aging in risk for AD, although the basis of this age-dependence is not established. Neuropathologically, the hallmarks of AD are deposition of extracellular amyloid plaques that are predominantly composed of amyloid-β (Aβ) peptide and intracellular neurofibrillary tangles (NFTs) comprised of hyperphosphorylated microtubule-associated protein tau (2, 3). Other pathologic changes include synapse and neuron loss, and reactive gliosis (4).

Among these pathologies, the strongest correlate of cognitive impairment in individuals with AD is loss of synapses across neocortical regions (5, 6), with excitatory synapses onto dendritic spines particularly affected (7, 8). Substantial evidence now indicates that aggregation of Aβ into soluble oligomers is a primary source of synaptotoxicity in AD (9–14). Although studies continue to elucidate how Aβ acts to eliminate dendritic spines, there is evidence that soluble Aβ inhibits mechanisms of long-term potentiation and/or engages mechanisms of long-term depression, including reducing NMDA receptor dependent Ca^2+^ influx, mGlutamate receptor (mGluR) activation, and low level caspase-3 activation (10, 12). The final common mechanism for these pathways converge on altered endocytotic recycling of GluR, resulting in reduced synaptic expression of GluR1 and GluR2 containing AMPA receptors and synaptic NMDA receptor (15).

Recently, several studies have identified visinin-like protein 1 (Vilip1), a protein encoded by the visinin-like 1 (VSNL1) gene, as a biomarker of AD. Vilip1 concentrations in cerebrospinal fluid and plasma are elevated in AD subjects relative to normal controls (16, 17) and to non-AD dementia subjects (17). Higher levels of cerebrospinal fluid Vilip1 also predicted a faster rate of cognitive decline (18). Other biomarkers for AD, such as cerebrospinal fluid measures of Aβ, tau, and phospho-tau (19), are strongly implicated in the pathogenesis of AD by genetic, post-mortem, animal model, and *in vitro* studies. To date, the evidence for Vilip1 is much more limited. We reported that genetic variations in VSNL1 were associated with risk for psychosis in AD (20), a phenotype characterized by more rapid cognitive deterioration than seen in AD subjects without psychosis (21, 22). Qualitative studies have reported that Vilip1 can be detected in association with neuritic plaques and NFTs in neocortex of AD subjects (23), and may contribute to phosphorylation of tau and Ca^2+^-mediated cell death (24).

Vilip1 is a highly brain expressed member of the visinin-like protein subfamily of neuronal calcium sensors (25). Vilip1, like other subfamily members appears to modify receptor recycling (26). For example, the closely related subfamily member, hippocalcin, is necessary for NMDA receptor dependent long-term depression via GluR endocytosis (27, 28). Whether Vilip1 has effects on synaptic plasticity processes implicated in synapse loss in AD, such as GluR recycling, long-term potentiation, and long-term depression, is not known. However, Vilip1 has a higher affinity for Ca^2+^ than calmodulin, suggesting it may respond to the lower Ca^2+^ levels, which induce long-term depression (25).

The above findings are consistent with the hypothesis that VSNL1/Vilip1 may contribute to the risk for AD, possibly via age-dependent alterations in expression or by affecting processes that contribute to synapse or neuronal loss. To date, however, very little is known about normal VSNL1 expression in brain, whether it is modulated by genetic variation, and the brain-related biologic co-expression networks in which VSNL1 participates. To begin to address these questions, we assessed VSNL1 expression in two regions of frontal cortex obtained from 209 subjects spanning the adult age range, and without evidence of psychiatric or neurodegenerative illness. We found that VSNL1 expression was present throughout the adult life span and was unaffected by age, sex, and common genetic variants in cis and trans. VSNL1 co-expression networks included KEGG pathways for calcium signaling, AD, and pathways implicated in synaptic pathology in AD.

## Materials and Methods

### Subjects

All of the brain specimens were collected during autopsies conducted at the Allegheny County Office of the Medical Examiner with permission obtained from the subjects’ next-of-kin. The protocol used to obtain consent was approved by the University of Pittsburgh Institutional Review Board (IRB) and Committee for Oversight of Research Involving the Dead. An independent committee of experienced clinicians made consensus DSM-IV diagnoses for each subject, using information obtained from clinical records and structured interviews with surviving relatives. These procedures were IRB approved. Samples from a total of 212 subjects without any DSM-IV diagnosis (i.e., including no diagnosis of a cognitive disorder) were obtained for use in this study.

### Tissue processing

Upon brain collection, ~2 cm coronal blocks from the right hemisphere were cut through the rostro-caudal extent of the brain and stored at −80°C. The RNA integrity (RIN) of each brain was assessed by chromatography (Agilent Bioanalyzer, Santa Clara, CA, USA). Samples were obtained from two prefrontal cortex (PFC) regions: Brodmann areas (BA) 11 and 47. These areas were selected based on prior findings showing robust age-related changes in gene expression that were highly correlated with other PFC regions (e.g., BA9) (29). Gray matter samples containing all six layers and excluding white matter were harvested from three to four consecutive 20 μm sections and stored in Trizol reagent.

### RNA arrays

Total RNA was extracted from frozen BA11 and BA47 samples stored in TRIZOL and were processed for microarray analysis using GeneChip Human Gene 1.1 ST from Affymetrix according to manufacturer’s protocol (http://www.affymetrix.com). Gene expression data were extracted using Expression Console build 1.2.1.20. The normalization method is based on quantile normalization to eliminate batch effects. Data from arrays were processed by RMA method. Gene expression probes were processed at gene-level and taken in log2 scale for further analysis. After normalization, 33,297 gene-level probes remained. Three samples were removed from study, one due to poor array quality, one due to outlier effect, and one due to XXY genotype, resulting in a final sample size of 209 subjects (Table 1).

**Table 1:** **Demographic and technical characteristics of human subjects**.

Variable	*N* (%) or mean (SD)
Age (years)	50.5 (14.6)
Range	16–91
Sex
Male	166 (79)
Female	43 (21)
Race
Caucasian	178 (85)
African-American	31 (15)
PMI	17.2 (5.9)
Range	4.8–37.5
pH	6.7 (0.3)
Range	5.8–7.6
RIN	8.0 (0.73)
Range	5.9–9.6

*PMI, post-mortem interval; RIN, RNA integrity number*.

### Genotyping

DNA samples for 204 of the subjects were available, and processed on Affymetrix SNP 6.0 arrays, assessing genotype at 906,600 SNPs. Genotype calls were generated using Affymetrix Genotyping Console version 4.1.3. For intensity quality control (QC), we used Contrast QC, which is the per sample QC test in the Affymetrix SNP 6.0 intensity array; two samples were removed after QC, leaving a final sample of 202 subjects for genotype measures. Birdseed v2 algorithm was used for genotyping, using the EM algorithm to drive a maximum likelihood fit of a two dimensional Gaussian mixture model.

### Statistical analysis

#### Age-dependent expression

Linear regression models were fitted to assess the association between VSNL1 expression level and demographic factors (age, sex, race). These models included as covariates the technical factors brain pH and RIN, as both were significantly associated with VSNL1 expression: *p* = 7.21e-09 (BA11) and 1.94e-04 (BA47) for pH association and *p* = 3.66e-12 (BA11) and 1.16e-08 (BA47) for RIN association. There was no significant association of VSNL1 expression with post-mortem interval (PMI, *p* = 0.26 and 0.36 in BA11 and BA47, respectively) and thus PMI was not included in the models.

#### eQTL mapping

eQTL analysis was performed in the 169 Caucasian subjects for whom genotype data were available. PCA analysis did not indicate population substructure within this cohort. All subjects had call rates >98%. SNPs were filtered out using the following criteria: (i) sample missing rate >10%, (ii) Minor allele frequency (MAF) <5%, and (iii) *p* value of Hardy–Weinberg equilibrium (HWE) test <10^−3^. SNPs were defined as in cis if they were located within 50 kbp of VSNL1. All other SNPs were defined as in trans.

The eQTL model adjusted for age, pH, and RIN values (each was significantly associated with gene expression in both BA11 and BA47) since the effects of those covariates may confound eQTL findings. The eQTL model with three covariates for a given genotype was:

$$G_{\mathrm{ij}}=\alpha_{i}+\gamma_{i}X_{j}+\sum_{k=1}^{3}\beta_{\mathrm{ik}}S_{\mathrm{jk}}+\in_{\mathrm{ij}}$$

in which: *G_ij_*: gene expression of gene *i* of subject *j*; α_*i*_: intercept term of gene *i*; γ*_i_*: effect of the selected genotype to gene *i* based on additive model; *X_j_*: genotype of subject *i*, 0 (homozygous major alleles), 1 (heterozygous calls), and 2 (homozygous minor alleles); β*_ik_*: the effect of covariates *k*; *k* = 1 (age), 2 (pH), and 3 (RIN) in gene *i*; *S_jk_*: the value of covariate *k* of subject *j*; *k* = 1 (age), 2 (pH), and 3 (RIN);

$$\in_{\mathrm{ij}}∼N\left(0,1\right)$$

We used the “Matrix eQTL” R package, a recent computationally efficient package for eQTL analysis (30), to detect the desired trans-eQTLs. We applied the adaptive weighted (AW) Fisher’s method (31) for meta-analysis to combine eQTL *p*-values from two brain regions (BA11 and BA47). The AW Fisher’s method has the advantage to distinguish study homogeneity and heterogeneity by assigning 0 or 1 study (brain region) weights for each eQTL. Detected eQTLs from the meta-analysis can have three possible resulting weights: (1,1) meaning detected eQTL in both brain regions; (1,0) showing detected eQTL in BA11 but not in BA47 and vice versa for (0,1) weight. In order to avoid heterogeneity of eQTL findings across brain regions, we selected only eQTLs that were identified in both brain regions with (1,1) weights in the AW Fisher’s method.

#### Co-expression pathway analyses

To reveal the co-expression structure of VSNL1, we selected the top 400 positively and negatively correlated genes in both brain regions. The minimum observed correlation among the selected genes was 0.64, and the associated *p*-values suggest the corresponding false discovery rate (FDR) is <1e-04. Pathway analysis was performed via over representation analysis based on Fisher’s exact test using pathway information retrieved from KEGG, BIOCARTA, and REACTOME databases. The Benjamini Hochberg procedure was used to control FDR for pathways (32).

## Results

### VSNL1 expression

**Figure 1:**
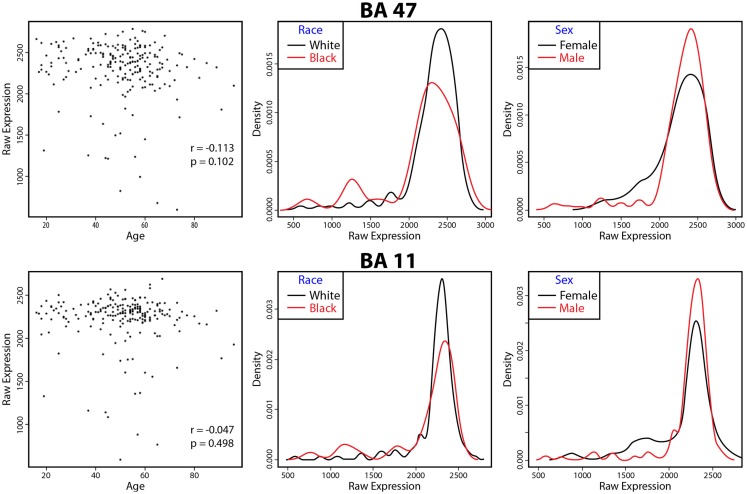
**VSNL1 expression as a function of age, race, and sex**. The correlation of VSNL1 expression levels with age are shown in the first panels for each area. The second and third panels show the density of expression levels as a function of race and sex. No associations were significant. BA, Brodmann area.

There were no significant associations of VSNL1 expression in either BA11 or BA47 with age, race, or gender (Figure 1).

### VSNL1 eQTL analysis

**Figure 2:**
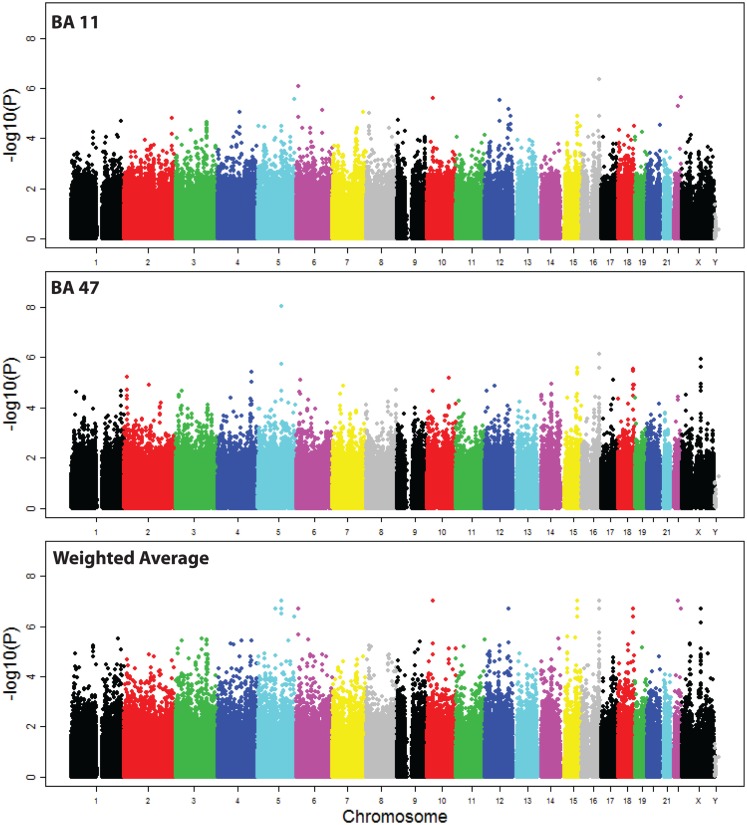
**Manhattan plot of SNP associations with VSNL1 expression**. Associations are shown for each region, and for the weighted average of the two regions in the meta-analysis. BA, Brodmann area.

There was no significant association of VSNL1 expression with 40 genotyped SNPs located in the cis-regulatory region (Table S1 in Supplementary Material). Examination of the association of VSNL1 expression with SNPs in trans revealed 27 SNPs with suggestive evidence of association (*p* < 10^−6^, Figure 2; Table S2 in Supplementary Material). However, no SNPs reached the threshold for genome wide significance (*p* < 10^−8^).

### VSNL1 co-expression networks

We next evaluated VSNL1 co-expression. Although VSNL1 expression was itself not age dependent, many of its potential co-expression partners show age-dependent alterations in expression, including genes related to neurodegeneration (29, 33). Therefore, we separately evaluated co-expression in subjects under and over 50 years of age. The 400 genes with the greatest positive and negative correlations with VSNL1 expression in each age group are shown in Table S3 in Supplementary Material.

The KEGG, BIOCARTA, and REACTOME pathways showing the most significant loading for genes positively and negatively correlated with VSNL1 expression are shown in Tables 2 and 3, respectively (all pathways are shown in Table S4 in Supplementary Material). In subjects under 50, VSNL1 was positively correlated with genes in the KEGG pathway for AD and the BIOCARTA pathway for P35 signaling in AD (P35Alzheimers). The association with the KEGG pathway was driven by correlations with amyloid precursor protein (APP), ATP2A2, CALM1, CDK5, CHP, GSK3B, ITPR1, NDUFAB1, NDUFV2, PPP3CA, PPP3CB, and UQCRH. The association with the P35Alzheimers pathway was driven by three of the same genes (APP, CDK5, and GSK3B) as well as PPP2CA. In subjects over 50, the co-expression of VSNL1 with these AD pathways was weakened, with CDK5, CHP, and NDUFAB1 no longer among the top co-expressed genes.

**Table 2:** **Top pathways identified by genes showing positive correlations with VSNL1 expression**.

Pathway	Age under 50			Age over 50		
	BA 47	BA 11	AVG	BA 47	BA 11	AVG
**KEGG**						
Aldosterone regulated sodium reabsorption	5.63E−03	5.00E−03	1.47E−05	9.00E−03	7.44E−03	1.96E−05
Alzheimers disease	1.20E−04	1.43E−03	1.47E−05	5.29E−03	6.55E−02	3.52E−04
Calcium signaling pathway	4.83E−04	1.08E−04	1.47E−05	1.10E−04	5.21E−03	1.96E−05
Cardiac muscle contraction	9.35E−04	6.19E−04	1.47E−05	8.94E−04	1.34E−03	1.96E−05
Epithelial cell signaling in *Helicobacter pylori* infection	1.96E−03	1.78E−05	1.47E−05	3.69E−04	2.02E−04	1.96E−05
Long-term potentiation	1.35E−03	1.04E−03	1.47E−05	2.92E−04	6.36E−03	1.96E−05
Oocyte meiosis	2.23E−04	9.68E−06	1.47E−05	2.76E−03	1.17E−03	1.96E−05
Oxidative phosphorylation	2.59E−04	1.59E−04	1.47E−05	7.01E−03	7.93E−03	1.96E−05
Proximal tubule bicarbonate reclamation	4.52E−03	3.60E−03	1.47E−05	5.72E−03	6.82E−03	1.96E−05
*Vibrio cholerae* infection	1.20E−04	2.10E−06	1.47E−05	8.76E−05	3.23E−06	1.96E−05
WNT signaling pathway	2.61E−03	1.70E−04	1.47E−05	2.88E−04	9.06E−03	1.96E−05
**BIOCARTA**						
AKAP centrosome pathway	1.19E−02	1.30E−02	2.58E−04	1.87E−03	1.67E−02	1.96E−05
CDC42RAC pathway	3.70E−03	2.56E−02	7.89E−05	4.59E−01	1.48E−01	1.13E−01
CHREBP2 pathway	1.17E−03	4.42E−03	1.47E−05	1.10E−03	1.64E−03	1.96E−05
CK1 pathway	5.35E−04	3.60E−03	1.47E−05	4.84E−04	3.06E−02	1.96E−05
CREB pathway	4.45E−03	4.35E−03	1.47E−05	5.62E−03	6.82E−03	1.96E−05
FCER1 pathway	7.74E−03	8.12E−03	2.74E−05	1.01E−02	1.27E−02	1.03E−04
GPCR pathway	1.20E−04	1.67E−03	1.47E−05	3.44E−04	1.27E−02	1.96E−05
HDAC pathway	3.18E−03	3.16E−03	1.47E−05	5.00E−04	4.19E−03	1.96E−05
MEF2D pathway	5.35E−04	2.56E−02	1.47E−05	3.20E−02	3.06E−02	8.39E−04
NDKDYNAMIN pathway	4.52E−03	5.83E−04	1.47E−05	3.73E−02	1.17E−03	1.96E−05
NFAT pathway	5.02E−04	1.74E−03	1.47E−05	1.88E−05	2.67E−03	1.96E−05
NOS1 pathway	3.92E−05	1.04E−03	1.47E−05	1.39E−04	1.80E−03	1.96E−05
P35Alzheimers pathway	5.30E−03	5.45E−03	1.47E−05	5.71E−02	5.43E−02	3.92E−03
PGC1A pathway	1.94E−03	1.74E−03	1.47E−05	4.36E−05	2.67E−03	1.96E−05
VIP pathway	4.04E−04	1.44E−02	1.47E−05	3.15E−03	1.78E−02	1.96E−05
**REACTOME**						
Acetylcholine neurotransmitter release cycle	2.67E−03	1.54E−04	1.47E−05	1.93E−04	4.13E−04	1.96E−05
DARPP32 events	3.92E−05	7.66E−05	1.47E−05	1.02E−04	1.17E−03	1.96E−05
Dopamine neurotransmitter release cycle	7.12E−03	3.91E−06	1.47E−05	8.76E−05	4.97E−06	1.96E−05
Formation of tubulin folding intermediates by CCT TRIC	1.94E−03	8.67E−03	1.47E−05	1.07E−02	1.19E−02	1.03E−04
Glucose regulation of insulin secretion	1.13E−03	6.15E−03	1.47E−05	9.88E−02	3.19E−01	4.45E−02
Glutamate neurotransmitter release cycle	2.31E−03	1.84E−04	1.47E−05	2.68E−04	4.37E−04	1.96E−05
Integration of energy metabolism	1.33E−04	3.60E−03	1.47E−05	2.34E−02	1.57E−01	4.65E−03
Neurotransmitter receptor binding and downstream transmission in the postsynaptic cell	1.94E−03	2.33E−05	1.47E−05	5.00E−04	2.67E−03	1.96E−05
Neurotransmitter release cycle	5.40E−03	9.68E−06	1.47E−05	1.02E−04	1.15E−05	1.96E−05
Norepinephrine neurotransmitter release cycle	3.89E−03	2.60E−04	1.47E−05	3.44E−04	5.25E−04	1.96E−05
Opioid signaling	1.13E−04	1.44E−04	1.47E−05	3.37E−06	1.30E−03	1.96E−05
PLC beta-mediated events	2.11E−03	4.30E−02	6.76E−05	3.44E−04	5.43E−02	1.96E−05
PLC gamma1 signaling	4.39E−04	9.35E−03	1.47E−05	3.44E−04	1.27E−02	1.96E−05
Regulation of insulin secretion	2.74E−04	3.32E−03	1.47E−05	4.72E−03	9.62E−02	4.34E−04
Regulation of insulin secretion by acetylcholine	5.40E−03	5.66E−03	1.47E−05	1.02E−04	1.00E−02	1.96E−05
Regulation of insulin secretion by free fatty acids	3.70E−03	2.88E−03	1.47E−05	7.73E−05	6.82E−03	1.96E−05
Regulation of insulin secretion by glucagon like peptide 1	1.94E−03	6.08E−03	1.47E−05	9.99E−05	2.67E−03	1.96E−05
Regulation of ornithine decarboxylase	7.81E−04	1.38E−01	7.89E−05	1.51E−01	1.50E−02	2.74E−03
Serotonin neurotransmitter release cycle	7.12E−03	3.91E−06	1.47E−05	8.76E−05	4.97E−06	1.96E−05
Signaling by WNT	8.24E−04	1.00E−02	1.47E−05	4.25E−02	4.45E−03	1.03E−04
Signaling by NGF	2.25E−03	9.51E−04	1.47E−05	6.17E−03	1.41E−03	1.96E−05
Trafficking of AMPA receptors	3.27E−02	1.82E−04	1.47E−05	4.20E−02	2.30E−03	1.96E−05
Trafficking of GLUR2 containing AMPA receptors	1.07E−01	2.70E−04	2.74E−05	1.31E−01	4.56E−03	4.62E−04
Transmission across chemical synapses	5.94E−06	2.29E−11	1.47E−05	1.66E−08	1.53E−08	1.96E−05

*Q values are shown for each region, and for the weighted average (AVG) of the two regions in the meta-analysis*.

*BA, Brodmann area*.

**Table 3:** **Top pathways identified by genes showing negative correlations with VSNL1 expression**.

Pathway	Age under 50			Age over 50		
	BA 47	BA 11	AVG	BA 47	BA 11	AVG
**KEGG**						
Allograft rejection	1.00E+00	1.00E+00	1.00E+00	1.38E−01	5.40E−01	3.37E−02
Autoimmune thyroid disease	1.00E+00	1.00E+00	1.00E+00	1.38E−01	5.40E−01	3.37E−02
Cell adhesion molecules CAMS	2.23E−01	8.54E−01	6.73E−02	4.19E−02	4.75E−01	2.78E−03
Cytokine–cytokine receptor interaction	2.23E−01	5.94E−01	4.17E−02	4.19E−02	1.68E−01	1.20E−03
Focal adhesion	6.69E−02	1.00E+00	3.85E−02	2.50E−01	1.00E+00	1.88E−01
Graft versus host disease	1.00E+00	1.00E+00	1.00E+00	1.38E−01	5.40E−01	3.37E−02
NOTCH signaling pathway	2.98E−01	1.00E+00	3.27E−01	1.38E−01	7.50E−01	4.69E−02
Pathways in cancer	8.23E−01	1.00E+00	4.25E−01	9.59E−02	6.10E−01	1.92E−02
Propanoate metabolism	3.54E−01	1.00E+00	4.30E−01	1.49E−01	4.75E−01	3.37E−02
Proximal tubule bicarbonate reclamation	1.83E−01	6.41E−01	3.85E−02	1.49E−01	2.43E−01	9.36E−03
Regulation of actin cytoskeleton	6.05E−02	8.29E−01	1.57E−02	3.32E−01	1.00E+00	2.25E−01
**BIOCARTA**						
MCALPAIN pathway	9.06E−02	1.00E+00	4.83E−02	1.91E−01	1.00E+00	1.61E−01
**REACTOME**						
Ethanol oxidation	1.00E+00	1.00E+00	1.00E+00	2.79E−01	4.19E−01	4.69E−02
GAP junction trafficking	1.59E−01	5.29E−01	1.57E−02	8.51E−01	5.35E−01	2.85E−01
Immunoregulatory interactions between a lymphoid and a non-lymphoid cell	3.51E−01	1.00E+00	2.02E−01	7.45E−02	4.67E−01	5.83E−03
Integrin cell surface interactions	9.06E−02	5.29E−01	1.00E−02	1.73E−01	9.45E−01	8.26E−02
NOTCH HLH transcription pathway	9.06E−02	1.00E+00	6.51E−02	2.78E−01	4.11E−01	4.48E−02

*Q values are shown for each region, and for the weighted average (AVG) of the two regions in the meta-analysis*.

*BA, Brodmann area*.

Other neuronal calcium sensor family members have been shown to mediate processes dependent on glutamate receptor trafficking, such as long-term potentiation and long-term depression (27, 28, 34). Although the protein product of VSNL1, Vilip1, is known to alter availability of α4,β2-nicotinic acetylcholine receptors at the cell membrane via affects on endocytic trafficking (35), a similar effect on glutamate receptors has not been shown. It is of some interest, therefore, that among the top pathways identified by genes positively correlated with VSNL1 expression are the KEGG pathway for long-term potentiation and REACTOME pathways for trafficking of AMPA receptors. These associations include positive correlations with several AMPA receptor subunits: GRIA1, GRIA2, and GRIA3. Although not among the top pathways identified, there was also a significant positive correlation of VSNL1 expression with the KEGG pathway for long-term depression in both age groups (Table S4 in Supplementary Material).

## Discussion

We evaluated the hypothesis that VSNL1 may contribute to the development of AD by assessing whether VSNL1 demonstrated age-dependent expression and by determining the brain-related biologic co-expression networks in which VSNL1 participates. VSNL1 expression was present throughout the adult life span, but was not correlated with subject age. VSNL1 co-expression networks included AD pathways and pathways implicated in synaptic pathology in AD, including long-term potentiation, long-term depression, and trafficking of AMPA receptors. These latter findings provide an unbiased link in support of the hypothesis that VSNL1/Vilip1 may participate in molecular mechanisms of AD.

We found that VSNL1 is expressed throughout the adult lifespan in human frontal cortex and is independent of subject age. We, and others, have previously reported that there is a substantial overlap between genes demonstrating age-related changes in expression and genes involved in the pathogenesis of neurodegenerative illnesses, including AD (36). The lack of changes in VSNL1 expression with age in our subjects suggests that any contribution of VSNL1 to the development of AD is not via this age-dependent mechanism. However, this does not preclude that VSNL1 expression could vary with age in other brain regions relevant to AD pathogenesis, such as the hippocampus. Also, it remains possible that post-transcriptional processing of VSNL1 into Vilip1 protein may vary with age. Evaluating that alternative will require future study of brain Vilip1 levels in an aging cohort.

We had previously identified an association between genetic variation in VSNL1 and the psychotic phenotype of AD (20). Because one important mechanism by which genetic variants may affect risk for neurodegenerative disease is to alter the transcription of their gene products in brain (37), we evaluated whether VSNL1 expression in frontal cortex is associated within or nearby the VSNL1 locus. We did not detect an association of VSNL1 expression with any of the tested SNPs. However, the possibility that nearby SNPs, other than the ones tested, affect VSNL1 expression cannot be excluded. To assess this likelihood, we used data from the 1000 Genomes project to estimate what proportion of all common variants (minor allele frequency ≥5%) in VSNL1 were correlated with the SNPs tested in our analysis. Only 70% of the common variants were tagged by one or more of our SNPs with an *r*^2^ ≥ 0.8. The 30% of poorly tagged common variants includes the most strongly-associated SNP in our GWAS, rs4038131. It is also possible that genetic variants, including those evaluated in the current study, may alter VSNL1 expression only in the presence of neurodegenerative pathology. However, it should be noted that recent tests of SNPs in the AD risk genes ABCA7, BIN1, CD2AP, CD33, CLU, CR1, EPHA1, MS4A6A, MS4A6E, and PICALM for associations with the expression of their respective genes in brain tissue from AD and healthy control subjects have been largely negative (38, 39).

Although the focus of our study was on risk for AD, genetic variants in VSNL1, altered brain VSNL1 mRNA expression, and altered brain levels of Vilip1 protein have been reported in schizophrenia (40–42). Of particular relevance to the current report was the finding that microRNA miR-181b, which is elevated in schizophrenia, can downregulate VSNL1 expression in model systems and correlates with reduced VSNL1 expression in the superior temporal gyrus in subjects with schizophrenia (40). We were able to replicate the negative correlation between VSNL1 expression and expression of pre-miR-181b genes in our normal aging cohort in both BA11 and BA47 (Figure S1 in Supplementary Material). However, how this normative regulation of VSNL1 by miR-181b might be changed in the presence of AD pathology and/or genetic variation in VSNL1, and in particular whether they may interact to underlie the association of VSNL1 genetic variation with psychosis in AD, is not known.

VSNL1 was co-expressed with genes in several distinct pathways. This included co-expression with genes identified with AD pathways and genes involved in mediating synaptic plasticity mechanisms such as long-term potentiation, long-term depression, and trafficking of AMPA receptors. These findings support a role for Vilip1 in these processes, as co-expressed genes tend to be related functionally (43). However, the specific nature of any functional relationship cannot be readily inferred, as co-expression can arise from many sources (44). For example, gray matter homogenates are a mixture of many cell types. Multiple genes that are specifically enriched in a single cell type, e.g. microglia, may be identified as co-expressed (43, 45). Alternatively, genes may share a mechanism regulating their transcription such as a transcription factor in common (46), proximity within the linear sequence of DNA leading to synchronous transcription (47), or colocalization within the spatial configuration of chromosomes (48). Similarly, epigenetic control of transcription via histone acetylation and methylation can lead to co-expression of neighboring genes (49). Finally, mRNA degradation via microRNA binding may also lead multiple targets of a given microRNA to demonstrate correlated expression (50).

In summary, VSNL1 is co-expressed with functional groups and gene transcripts in AD pathways, including APP itself and pathways implicated in synaptic pathology in AD. These findings provide an unbiased link between VSNL1 and molecular mechanisms of AD. However, whether increased expression of APP may drive increased VSNL1 expression, increased VSNL1 expression drives increased APP expression, or both are concurrently altered downstream of another factor will need to be evaluated in model systems. For example, evaluation of VSNL1 expression in transgenic mouse models of AD, or evaluation of APP expression after knockdown of VSNL1 would be indicated. Similarly, whether altering VSNL1 expression modifies synaptic pathology in AD would benefit from testing within *in vitro* and/or genetic mouse models.

## Conflict of Interest Statement

Dr. Robert A. Sweet served as a consultant for Lilly, USA. Mr. Chien-Wei Lin, Mr. Lun-Ching Chang, Dr. George C. Tseng, Ms. Caitlin M. Kirkwood, and Dr. Etienne L. Sibille have no conflicts of interest or financial interests to declare. The Review Editor Konasale Prasad declares that, despite being affiliated to the same institution as authors Chien-Wei Lin, Lun-Ching Chang, George C.Tseng, Caitlin M. Kirkwood, Etienne L. Sibille and Robert A. Sweet, the review process was handled objectively and no conflict of interest exists.

## Supplementary Material

The Supplementary Material for this article can be found online at http://www.frontiersin.org/Journal/10.3389/fpsyt.2015.00030/abstract
