# Supplementary material for: VSNL1 Co-Expression Networks in Aging Include Calcium Signaling, Synaptic Plasticity, and Alzheimer’s Disease Pathways
Source: Front Psychiatry. 2015 Mar 9;6:30. doi: 10.3389/fpsyt.2015.00030 (PMC4353182; doi:10.3389/fpsyt.2015.00030)
Supplement: Supplementary file 1 [file table_1.docx]

**Supplemental Table 1. Association of SNPs in cis with VSNL1 expression.** eQTL p-values are shown for each brain region, and for the weighted average (AVG) of the two regions in the meta-analysis. Chr- Chromosome; MAF- Minor Allele Frequency; BA- Brodmann Area.

| **Chr** | **Position** | **SNP** | **MAF** | **BA 11** | **BA 47** | **AVG** |
| --- | --- | --- | --- | --- | --- | --- |
| 2 | 17674793 | rs13020775 | 0.376 | 0.3772 | 0.1091 | 0.2447 |
| 2 | 17675735 | rs2030328 | 0.236 | 0.3071 | 0.7576 | 0.4135 |
| 2 | 17678048 | rs368430 | 0.25 | 0.671 | 0.7799 | 0.7941 |
| 2 | 17694045 | rs1519562 | 0.385 | 0.4598 | 0.1495 | 0.3474 |
| 2 | 17701234 | rs11885715 | 0.385 | 0.4598 | 0.1495 | 0.3474 |
| 2 | 17706978 | rs7572154 | 0.399 | 0.685 | 0.3085 | 0.6702 |
| 2 | 17712696 | rs446947 | 0.241 | 0.4605 | 0.7238 | 0.5877 |
| 2 | 17738199 | rs426590 | 0.241 | 0.4605 | 0.7238 | 0.5877 |
| 2 | 17769156 | rs12478492 | 0.332 | 0.3864 | 0.03703 | 0.1121 |
| 2 | 17770937 | rs12623114 | 0.376 | 0.7502 | 0.05782 | 0.254 |
| 2 | 17774634 | rs3109180 | 0.318 | 0.638 | 0.06817 | 0.2545 |
| 2 | 17774912 | rs4038130 | 0.0647 | 0.3608 | 0.9671 | 0.4767 |
| 2 | 17782556 | rs1426510 | 0.238 | 0.6262 | 0.7981 | 0.7537 |
| 2 | 17784986 | rs11096479 | 0.374 | 0.8385 | 0.04377 | 0.2257 |
| 2 | 17785380 | rs12053198 | 0.376 | 0.7502 | 0.05782 | 0.254 |
| 2 | 17785953 | rs10856770 | 0.438 | 0.8371 | 0.04565 | 0.2323 |
| 2 | 17789103 | rs12373666 | 0.435 | 0.746 | 0.03451 | 0.1745 |
| 2 | 17790442 | rs13006235 | 0.164 | 0.1424 | 0.5349 | 0.2049 |
| 2 | 17790513 | rs7567064 | 0.324 | 0.4868 | 0.04448 | 0.1535 |
| 2 | 17792215 | rs13019617 | 0.438 | 0.8371 | 0.04565 | 0.2323 |
| 2 | 17794902 | rs13011227 | 0.375 | 0.5788 | 0.06992 | 0.2419 |
| 2 | 17796165 | rs2001674 | 0.248 | 0.9367 | 0.6807 | 0.9746 |
| 2 | 17799288 | rs11683808 | 0.455 | 0.8627 | 0.1127 | 0.4335 |
| 2 | 17810994 | rs4077269 | 0.382 | 0.6572 | 0.07636 | 0.281 |
| 2 | 17814030 | rs2710684 | 0.182 | 0.1746 | 0.1203 | 0.15 |
| 2 | 17816326 | rs17380736 | 0.397 | 0.02948 | 0.7914 | 0.04576 |
| 2 | 17821056 | rs2710687 | 0.347 | 0.1125 | 0.9438 | 0.1643 |
| 2 | 17833639 | rs2555096 | 0.393 | 0.0329 | 0.942 | 0.05089 |
| 2 | 17834452 | rs6751113 | 0.397 | 0.03043 | 0.8958 | 0.04719 |
| 2 | 17834754 | rs2555099 | 0.344 | 0.1791 | 0.6834 | 0.2534 |
| 2 | 17835064 | rs2555100 | 0.344 | 0.1791 | 0.6834 | 0.2534 |
| 2 | 17837741 | rs2710667 | 0.338 | 0.1518 | 0.8054 | 0.2174 |
| 2 | 17838396 | rs2710669 | 0.341 | 0.1519 | 0.7963 | 0.2175 |
| 2 | 17845296 | rs2710673 | 0.156 | 0.2175 | 0.1957 | 0.2506 |
| 2 | 17846162 | rs2555107 | 0.349 | 0.2191 | 0.6183 | 0.305 |
| 2 | 17846592 | rs2710674 | 0.156 | 0.2175 | 0.1957 | 0.2506 |
| 2 | 17849667 | rs11096482 | 0.403 | 0.411 | 0.4133 | 0.5335 |
| 2 | 17851241 | rs17315242 | 0.156 | 0.8078 | 0.8781 | 0.9035 |
| 2 | 17858469 | rs2555075 | 0.356 | 0.1382 | 0.6372 | 0.1992 |
| 2 | 17877105 | rs7572870 | 0.347 | 0.1648 | 0.6274 | 0.2348 |
