# Supplementary material for: VSNL1 Co-Expression Networks in Aging Include Calcium Signaling, Synaptic Plasticity, and Alzheimer’s Disease Pathways
Source: Front Psychiatry. 2015 Mar 9;6:30. doi: 10.3389/fpsyt.2015.00030 (PMC4353182; doi:10.3389/fpsyt.2015.00030)
Supplement: Supplementary file 2 [file table_2.docx]

**Supplemental Table 2. SNPs in trans showing suggestive association with VSNL1 expression (p < 10^-6^).** eQTL p-values are shown for each brain region, and for the weighted average (AVG) of the two regions in the meta-analysis. Chr- Chromosome; MAF- Minor Allele Frequency; BA- Brodmann Area.

| **Chr** | **Position** | **SNP** | **MAF** | **BA 11** | **BA 47** | **AVG** |
| --- | --- | --- | --- | --- | --- | --- |
| 5 | 81172212 | rs6452434 | 0.05 | 9.88E-05 | 5.74E-05 | 2.00E-07 |
| 5 | 111605951 | rs13361927 | 0.0794 | 3.23E-05 | 2.17E-05 | 2.00E-07 |
| 5 | 111620915 | rs10068536 | 0.0824 | 1.43E-04 | 5.11E-05 | 3.00E-07 |
| 5 | 111788620 | rs255720 | 0.0533 | 7.41E-04 | 8.89E-09 | 1.00E-07 |
| 5 | 111793954 | rs163622 | 0.0769 | 4.94E-05 | 1.91E-06 | 1.00E-07 |
| 5 | 169813200 | rs186385 | 0.0824 | 2.80E-06 | 4.47E-03 | 4.00E-07 |
| 6 | 11874298 | rs7740058 | 0.132 | 8.20E-07 | 7.72E-04 | 2.00E-07 |
| 10 | 26231850 | rs7070437 | 0.0964 | 2.48E-06 | 2.13E-05 | 1.00E-07 |
| 12 | 115744758 | rs10507258 | 0.0529 | 6.87E-06 | 5.20E-04 | 2.00E-07 |
| 15 | 81339871 | rs1320322 | 0.0824 | 1.65E-04 | 5.18E-05 | 4.00E-07 |
| 15 | 81350739 | rs950426 | 0.0799 | 5.48E-05 | 2.89E-05 | 2.00E-07 |
| 15 | 81358042 | rs12907050 | 0.0735 | 2.53E-05 | 3.84E-06 | 1.00E-07 |
| 15 | 81360790 | rs12913347 | 0.0559 | 7.61E-05 | 2.67E-06 | 1.00E-07 |
| 15 | 81374451 | rs10519308 | 0.0765 | 1.30E-05 | 4.71E-06 | 1.00E-07 |
| 16 | 82128197 | rs2955163 | 0.0559 | 4.37E-07 | 2.67E-06 | 1.00E-07 |
| 16 | 82131578 | rs2955160 | 0.0618 | 1.24E-05 | 7.51E-05 | 2.00E-07 |
| 16 | 82225566 | rs1862819 | 0.294 | 1.49E-04 | 7.63E-07 | 1.00E-07 |
| 18 | 70477427 | rs10514055 | 0.134 | 1.34E-03 | 2.99E-06 | 2.00E-07 |
| 18 | 70477497 | rs10514056 | 0.135 | 8.03E-04 | 1.29E-05 | 4.00E-07 |
| 18 | 70484850 | rs17727236 | 0.126 | 6.49E-04 | 1.74E-05 | 4.00E-07 |
| 18 | 70484877 | rs17727260 | 0.121 | 2.75E-04 | 3.59E-06 | 2.00E-07 |
| 22 | 35390101 | rs7285304 | 0.0912 | 5.24E-06 | 5.03E-05 | 1.00E-07 |
| 22 | 35390306 | rs9610191 | 0.0882 | 5.40E-06 | 3.75E-05 | 1.00E-07 |
| 22 | 48460689 | rs17697394 | 0.182 | 2.34E-06 | 2.16E-03 | 2.00E-07 |
| X | 90482887 | rs2208213 | 0.115 | 2.82E-03 | 1.18E-06 | 2.00E-07 |
| X | 90483759 | rs4261049 | 0.115 | 2.82E-03 | 1.18E-06 | 2.00E-07 |
| X | 90539476 | rs1028556 | 0.115 | 6.61E-03 | 2.45E-06 | 7.00E-07 |
