# Supplementary figures and images for: VSNL1 Co-Expression Networks in Aging Include Calcium Signaling, Synaptic Plasticity, and Alzheimer’s Disease Pathways
Source: Front Psychiatry. 2015 Mar 9;6:30. doi: 10.3389/fpsyt.2015.00030 (PMC4353182; doi:10.3389/fpsyt.2015.00030)

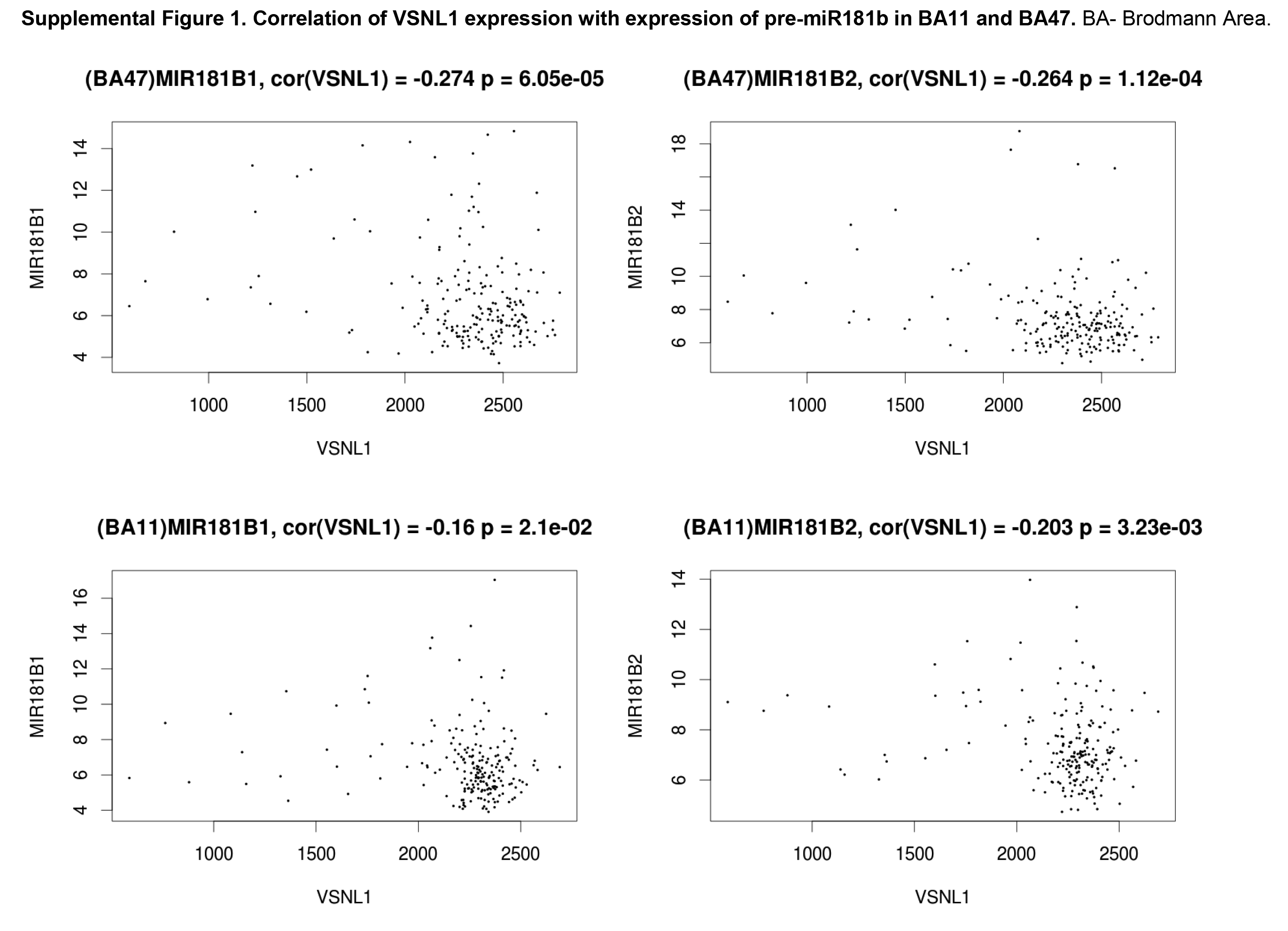

Supplement: Supplementary file 5 [file image_1.tif]
